# Salivary Biomarkers for Diagnosis and Therapy Monitoring in Patients with Heart Failure. A Systematic Review

**DOI:** 10.3390/diagnostics11050824

**Published:** 2021-05-02

**Authors:** Aidonis Rammos, Aris Bechlioulis, Petros Kalogeras, Evanthia E. Tripoliti, Yorgos Goletsis, Anna Kalivi, Effrosyni Blathra, Pietro Salvo, M. Giovanna Trivella, Tommaso Lomonaco, Roger Fuoco, Francesca Bellagambi, Chris J. Watson, Abdelhamid Errachid, Dimitrios I. Fotiadis, Lampros K. Michalis, Katerina K. Naka

**Affiliations:** 1 Second Department of Cardiology, Faculty of Medicine, School of Health Sciences, University of Ioannina and University Hospital of Ioannina, GR 45500 Ioannina, Greece; aidrammos@yahoo.gr (A.R.); md02798@yahoo.gr (A.B.); pkalog90@yahoo.com (P.K.); annakalivi@yahoo.gr (A.K.); 5677efiblathra@gmail.com (E.B.); lamprosmihalis@gmail.com (L.K.M.); 2 Department of Biomedical Research, Institute of Molecular Biology and Biotechnology, FORTH, GR 45110 Ioannina, Greece; etripoliti@gmail.com (E.E.T.); goletsis@uoi.gr (Y.G.); dimitris.fotiadis30@gmail.com (D.I.F.); 3 Department of Economics, University of Ioannina, GR 45110 Ioannina, Greece; 4 Institute of Clinical Physiology, Italian National Research Council, Via G. Moruzzi 1, PI 56124 Pisa, Italy; pietro.salvo@ifc.cnr.it (P.S.); trivella@ifc.cnr.it (M.G.T.); 5 Department of Chemistry and Industrial Chemistry, University of Pisa, PI 56124 Pisa, Italy; tommaso.lomonaco@unipi.it (T.L.); roger.fuoco@unipi.it (R.F.); francesca.bellagambi@univ-lyon1.fr (F.B.); 6 Institute of Analytical Sciences (ISA)—UMR 5280, University Claude Bernard Lyon 1, 69100 Lyon, France; abdelhamid.errachid-el-salhi@univ-lyon1.fr; 7 UCD Conway Institute, School of Medicine, University College Dublin, DUBLIN 4, Dublin, Ireland; chris.watson@qub.ac.uk; 8 Wellcome-Wolfson Institute for Experimental Medicine, Queen’s University Belfast, Belfast BT97BL, UK; 9 Unit of Medical Technology and Intelligent Information Systems, University of Ioannina, GR 45110 Ioannina, Greece

**Keywords:** biomarkers, heart failure, saliva, diagnosis, therapy monitoring

## Abstract

The aim of this study was to perform a systematic review on the potential value of saliva biomarkers in the diagnosis, management and prognosis of heart failure (HF). The correlation between saliva and plasma values of these biomarkers was also studied. PubMed was searched to collect relevant literature, i.e., case-control, cross-sectional studies that either compared the values of salivary biomarkers among healthy subjects and HF patients, or investigated their role in risk stratification and prognosis in HF patients. No randomized control trials were included. The search ended on 31st of December 2020. A total of 15 studies met the inclusion criteria. 18 salivary biomarkers were analyzed and the levels of all biomarkers studied were found to be higher in HF patients compared to controls, except for amylase, sodium, and chloride that had smaller saliva concentrations in HF patients. Natriuretic peptides are the most commonly used plasma biomarkers in the management of HF. Their saliva levels show promising results, although the correlation of saliva to plasma values is weakened in higher plasma values. In most of the publications, differences in biomarker levels between HF patients and controls were found to be statistically significant. Due to the small number of patients included, larger studies need to be conducted in order to facilitate the use of saliva biomarkers in clinical practice.

## 1. Introduction

Heart Failure (HF) is a clinical syndrome characterized by symptoms such as dyspnea or fatigue on exertion or at rest, and clinical signs (i.e., lower extremity oedema, elevated jugular venous pressure, pulmonary crackles, etc.) caused by a structural and/or functional cardiac abnormality, ultimately leading to reduced cardiac output [1]. HF is one of the leading causes of morbidity and mortality worldwide, with a prevalence ranging from 6% to 13% [2]. An analysis in 2012 estimated that the global cost of HF was $108 billion per year, of which $65 billon was attributed to direct and $43 billion to indirect costs [3].

The etiology of HF is quite variable, with most common causes being coronary artery disease, hypertension, arrhythmias, valvular and structural abnormalities, toxic damage from recreational substance use and chemotherapy, immune, inflammatory and metabolic causes, infiltrative diseases, genetic abnormalities, etc. [1]. Many patients may have more than one causative factor for HF. The classification of HF syndromes currently used in clinical practice for the management of patients is based on the left ventricular ejection fraction (LVEF), rather than the etiology. Accordingly, patients are characterized as HF with preserved, mid-range and reduced LVEF (HFpEF, HFmrEF and HFrEF when LVEF is ≥50%, 40–49%, and <40%, respectively) [1].

Prognosis varies depending on the cause but is generally poor [4]. In patients with HFrEF, therapy is based on well-established algorithms supported by large, multi-center, randomized clinical trials that have shown increased survival and improved quality of life [1]. On the contrary, for patients with HFpEF (usually older patients with many co-morbidities) there is no clear evidence that specific medications or other therapeutic approaches may increase survival. The European Society of Cardiology has published guidelines and several consensus and position papers on HF management [1,5].

However, due to the variations in causative factors, presenting symptoms and therapeutic options, decisions on diagnosis and treatment of HF remain challenging. In everyday clinical practice, biomarkers play an important and expanding role in HF diagnosis, therapy monitoring and risk stratification. Plasma natriuretic peptides such as brain natriuretic peptide (BNP) and N-terminal fragment BNP (NT-proBNP) have already been established in the diagnostic algorithm of HF, while other serum biomarkers such as soluble interleukin 1 receptor-like 1 (ST2), galectin-3 (Gal-3), copeptin, adrenomedullin, high sensitivity troponin (hsTn), growth differentiation factor 15 (GDF-15), adiponectin, C-reactive protein (CRP) and neprilysin have been tested without definite evidence yet to recommend them for clinical practice [1,6,7,8,9,10,11,12,13,14,15,16,17].

As in many other chronic diseases, an important goal in HF research is to identify biomarkers that could improve accuracy in diagnosis and monitoring. Another important issue is to detect biomarkers that could be measured in other biological fluids (instead of blood), because this procedure would be less invasive and easier to accomplish in all clinical settings. Towards that direction, point-of-care devices that can be used for HF monitoring outside the hospital setting, in primary health care or at home have been developed [8,18,19].

During the last years, saliva has emerged as a body fluid containing several proteins that could be used as potential biomarkers, with an important benefit that saliva can be easily collected with non-invasive procedures [20]. In addition, salivary diagnostics is currently catching onto the emerging field of Lab-on-Chip (LoC) and Point-of-Care (PoC) devices [20]. Saliva values of myoglobin, cardiac troponin I, creatine phosphokinase MB, myeloperoxidase, the natriuretic peptides (BNP and NT-proBNP), CRP, etc., have been used for the diagnosis of cardiovascular diseases in general and in most cases they have been found to correlate well with plasma concentrations [21,22,23,24,25]. On the other hand, very few have been tested specifically for HF diagnosis, monitoring and prognosis [26,27,28,29,30,31,32,33,34,35,36,37,38,39,40].

The current review aims to assess and summarize the potential role of salivary biomarkers in the diagnosis, progression monitoring and prognosis of patients with HF. The correlation between saliva and plasma values of these biomarkers was also studied.

## 2. Materials and Methods

This systematic review was performed according to the Preferred Reporting Items for Systematic Reviews and Meta-Analyses (PRISMA) 2020 guidelines [41].

### 2.1. Review Question

Which salivary biomarkers have been used for the diagnosis and therapy monitoring of patients with HF, and which of them provided a reliable means of disease detection?

### 2.2. Eligibility Criteria

Inclusion criteria for the search were case-control, cross-sectional studies that compared the values of one or more salivary biomarkers among healthy subjects and patients with HF. No randomized control trials were found in our search. Articles published before 1950, articles written in a language other than English, reviews, letters to the editors, and articles correlating salivary biomarkers with other diseases such as liver failure, cancer or periodontal diseases, were excluded.

### 2.3. Search Strategy

**Figure 1:**
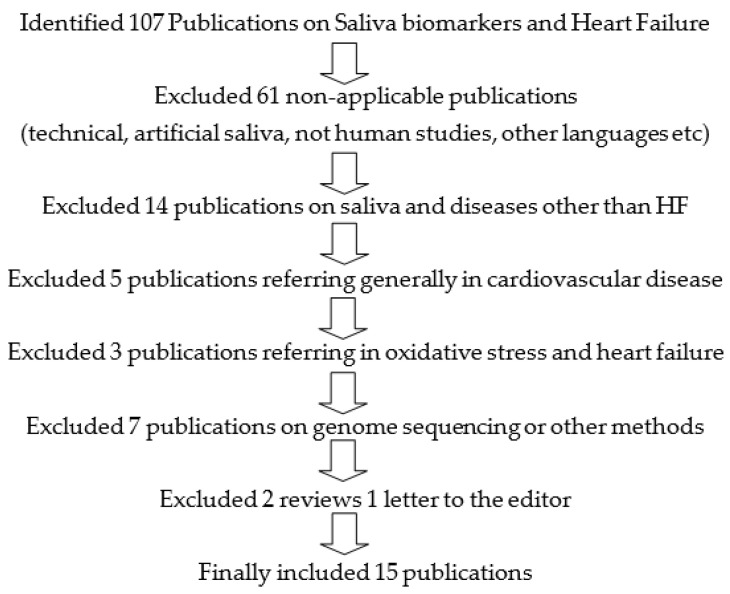
Search strategy on “Saliva Biomarkers and Heart Failure.

An advanced literature search was conducted using the following Mesh terms: (“Saliva” (Mesh) and “Biomarkers” (Mesh) or salivary biomarkers) and (“Heart Failure” (Mesh)). Study titles, abstracts, and full texts were extracted from PubMed until December 2020. After duplicates (22 in total) were removed, the search yielded a total of 107 titles. Titles were evaluated for relevance. From the relevant ones, the abstracts were reviewed and re-evaluated for relevance to our study question. Subsequently, the full text of accepted abstracts was reviewed. Of the full texts reviewed, those that did not contribute to our study question were excluded. Finally, the number of studies included in this review was 15 (Figure 1).

## 3. Results

Fifteen studies fulfilled the inclusion criteria [26,27,28,29,30,31,32,33,34,35,36,37,38,39,40]. Among these studies, eighteen separate salivary biomarkers were analyzed; salivary amylase, mainly its major form salivary alpha amylase (sAA), uric acid (UA), 8-isoprostaglandin F2α (8-isoPGF2α), lactate, galectin-3 (Gal-3), BNP, interleukin 6 (IL-6) and interleukin 10 (IL-10), CRP, protein S100-A7 (S10A7), cortisol, NT-proBNP, 8-epiprostaglandin F2α (8-epiPGF2α), endothelin, sodium, chloride, and potassium.

Only three studies measured salivary natriuretic peptides [29,30,35]. The first compared salivary NT-proBNP between controls (*n* = 40) and HF patients (*n* = 45). The salivary NT-proBNP concentrations from the healthy participants were below the limit of detection (LOD 16 pg/mL), while in HF patients salivary NT-proBNP ranged from 18.3 to 748.7 pg/mL with a median value of 76.8 pg/mL. The salivary NT-proBNP immunoassay showed sensitivity of 82.2% and specificity of 100%, positive predictive value of 100% and negative predictive value of 83.3%, with overall diagnostic accuracy at 90.6%. On the other hand, there was no correlation between salivary and plasma NT-proBNP concentrations in HF patients (R2 = 0.006, *p* = 0.66) [35]. The second study measured serum and salivary BNP from 75 hospitalized patients with HF, and also found no significant correlations between serum and salivary BNP (r = −0.064, *p* = 0.628), with a large positive bias of 480 pg/mL, indicating that serum concentrations of BNP were much higher than the salivary concentrations, and as serum BNP levels increased, the difference became larger [29]. In the third study, mean salivary BNP levels were higher in both hospitalised HF (*p* < 0.001) and outpatient HF patients (*p* = 0.02) compared to the control subjects (6.50 ng/L vs. 5.87 ng/L vs. 5.64 ng/L, respectively). A moderate correlation between salivary BNP and plasma NT-proBNP concentrations (*p* < 0.001, r = 0.459) was found [30]. Despite the rather small number of participants, all studies found higher natriuretic peptide levels in the saliva in HF patients compared to controls, but larger studies are needed to validate the clinical importance of the saliva values of natriuretic peptides as well as their correlation to plasma levels.

In chronic HF, inflammatory and neurohormonal activation takes place as a response to the failing heart. Activation of the renin-angiotensin system is responsible for the over-expression of the stress hormones, i.e., angiotensin-converting enzyme and angiotensin II. Cortisol has been implicated in the progress of chronic HF, since it binds to the mineralocorticoid receptor with an affinity equal to that of aldosterone and its pathophysiological role may be influenced by oxidative stress [42,43,44]. Analysis of saliva instead of serum is advantageous because salivary cortisol represents the unbound (i.e., free) hormone which is considered biologically active, while the vast majority of serum cortisol is bound to cortisol-binding globulin and albumin [45]. The number of patients included in these three studies is small (229 [32], 81 [34], and 27 [38], respectively). The LOD was 0.05 ng/mL, 0.07 ng/mL and 0.15 ng/mL respectively with values of HF patients ranging from 0.40–0.92 ng/mL, 0.19–0.55 ng/mL, and 6.88–8.33 ng/mL, respectively, in each study. Only in the third study were cortisol levels measured in controls, with values ranging from 5.43 to 6.88 ng/mL. Salivary cortisol was commonly increased in HF patients, while higher levels were associated with a reduced event-free interval and high evening levels were associated with increased mortality risk [32,34,38].

Galectin-3 (Gal-3) is a β-galactoside–binding lectin that plays a role in inflammatory and immune-mediated disorders. It is mainly expressed in activated macrophages and pathologically damaged cardiomyocytes and is considered as an active contributor to cardiac remodeling (including myocardial fibrogenesis) and development of HF. Gal-3 induces fibroblast proliferation and heterogeneous deposition of collagen types, eventually leading to loss of cardiac function [46,47]. Two studies have measured salivary Gal-3. In the first study, 105 HF patients (hospitalised or at routine outpatient visits) had a significantly higher cumulative risk of cardiovascular death or hospitalization when their salivary Gal-3 levels were higher than 172.58 ng/mL [28]. In the second study, samples from 63 HF patients were compared to healthy controls and were significantly elevated (both in saliva and serum), with a moderate correlation (r = 0.4, *p* < 0.01) between serum and salivary Gal-3 levels [33].

Almost all of the serum amylase activity in healthy adults is found at the pancreas and salivary glands [48]. Plasma amylase levels were found to be elevated in severe HF, possibly due to the mesenteric venous congestion and impaired peripheral tissue perfusion [49]. Two studies, with a small number of patients each, measured salivary amylase in HF patients. The first (which included 33 NYHA II and 17 NYHA III patients) found decreased salivary levels of amylase in the HF population; this was attributed to the impaired secretory function of salivary glands in HF patients that led to lower content and activity of salivary amylase compared to healthy controls [26]. The second study (which included 24 NYHA I-III patients and 24 controls) found no statistically significant difference between the control and HF groups in sAA levels, although there was a strong tendency for the morning values to be higher in HF patients, especially if measured within 30 min after awakening. It should be noted that there was a strong inter- and intra-subject variation and a small number of participants, while all HF patients were on b-blocker therapy that reduces sAA levels [36].

Apart from the activation of compensatory neurohormonal mechanisms, HF is also associated with hyper-lactatemia, oxidative stress, and hyperuricemia [27]. In one study, salivary lactate and 8-isoPGF2α from 44 patients with acute HF strongly correlated with serum NT-proBNP, while salivary uric acid did not. The LOD was 10 pg/mL and 6 µg/mL for 8-iso-PGF2α and lactate, respectively. Lactate levels positively correlated with NYHA class, while 8-isoprostaglandin F2α levels did not correlate to NYHA class [27]. In another study, salivary 8-epiPGF2α levels were significantly higher in patients with ischemic and dilated cardiomyopathy compared to controls and patients with coronary heart disease (*p* = 0.001). 8-epiPGF2α levels negatively correlated with LVEF and positively correlated with NYHA class [37]. Salivary UA was also examined by Klimiuk et al. [27] and was significantly higher in HF compared to healthy subjects in stimulated saliva. Moreover, in non-stimulated saliva the UA levels were higher in worse NYHA class patients.

C-reactive protein (CRP) is a phylogenetically highly conserved plasma protein that participates in the systemic response to inflammation. CRP and other inflammatory cytokines were examined as markers of HF severity and prognosis in the study by Dekker et al. [29]. Although serum and salivary levels of CRP were found to have a moderate correlation (r = 0.594, *p* < 0.001), salivary CRP levels were not associated with NYHA class [29]. In the same study in 75 hospitalized HF patients, a weak correlation for serum–salivary IL-6 (r = 0.288, *p* = 0.037), and no correlations for serum–salivary IL-10 (r = 0.068, *p* = 0.629) or serum–salivary BNP (r = −0.064, *p* = 0.628) were reported [29]. Interestingly, no biomarkers in this study were associated with NYHA class and only visible oral inflammation was found to be a significant predictor of HF severity. Two major classes of cytokines have also been implicated in HF, vasoconstrictor cytokines such as endothelin and vasodepressor proinflammatory cytokines such as Tumor Necrosis Factor-α (TNF-α), IL-6, and IL-1 [49,50]. These inflammatory mediators are expressed by all nucleated cell types residing in the myocardium, including the cardiac myocyte. In patients with HF, circulating as well as intra-cardiac levels of these cytokines have been reported to be elevated [51]. Salivary endothelin concentrations were raised two to six-fold in chronic HF patients vs. controls (*p* = 0.005), with a positive correlation to respective plasma concentrations (*p* = 0.032) and were able to detect chronic HF with 63% sensitivity 63% and 92% specificity. Endothelin salivary levels have also been shown to reflect symptom severity by NYHA class [39]. TNF-α has been studied extensively in periodontal inflammations and oral cancers and has been identified in detectable concentrations in human saliva with values correlating well to plasma values [52,53], indicating that salivary TNF-α could potentially serve as a HF biomarker, providing that supporting data emerged [54,55,56,57,58].

Mass spectrometry was used to analyze the saliva proteome in HF patients (*n* = 75) and healthy controls (*n* = 36), as reported by Zhang et al. Of the 728 proteins detected, only the protein S100-A7 (S10A7) was significantly different between saliva from NYHA III/IV HF patients compared to healthy controls. However, the underlying mechanisms relating S10A7 to the pathogenesis of HF are not clear and further investigations are needed [31].

White et al., in 1950, measured the saliva concentration of sodium, chloride, and potassium in a small number of patients with congestive HF (*n* = 27) and healthy subjects (*n* = 11). Congestive HF was found to be associated with lower sodium, lower chloride, and higher potassium mean concentrations in saliva than in controls, irrespectively of whether HF patients were on a low-salt or a regular diet. Although towards the right direction, i.e., lower sodium (congestion) and higher potassium (renal failure, etc.) in HF patients, there was no relationship in electrolyte concentrations between serum and saliva [40].

The studies fulfilling the eligibility criteria and their key conclusions are summarized in Table 1.

## 4. Discussion

A multitude of biomarkers have been proposed in the field of cardiovascular diseases in general and in HF more specifically, emphasized in one single in silico study which examined 161 serum or plasma biomarkers in HF [59]. This large variety of potential biomarkers studied reflects the complex pathophysiological mechanisms of HF, which involve inflammatory reactions, autonomic nervous system dysfunction, left ventricular remodeling (e.g., increased myocardial stiffness, decreased diastolic myocardial relaxation, increased left ventricular mass, decreased peak contractility), vascular alterations (e.g., increased arterial stiffening, decreased coronary flow reserve), and decreased mitochondrial response to the increased demand for adenosine triphosphate production; distinct biomarkers describe various pathways that have been proposed [60].

Despite the numerous biomarkers tested and standardized in blood specimens, this is not the case with saliva. There is an obvious lack of standardized protocols for biomarker determination in cardiovascular diseases and HF. Although the collection procedures are easy and not painful, saliva composition is affected by several variables, arising from variation of salivary flow rate, circadian rhythm, type of salivary stimulus, diet, age, physiological status, and methods of collection [61,62]. Another reason for the limited use of saliva diagnostics is the fact that levels of most biomarkers are lower in saliva than those found in serum [63]. Advances in analytical techniques are expected to make it feasible to measure multiple biomarkers using small volumes of biological fluids including saliva.

Most of the publications on saliva biomarkers in HF have been conducted during the last decade (11 out of 15 studies). The biomarkers have been tested in HF patients and compared to controls and/or different NYHA class patients. The age of participating patients ranged from 39 to 88 years old. Out of the 15 publications, seven included patients with HFrEF only, five with both HFpEF and HFrEF patients (without proving data separately for each phenotype), while three studies did not provide that information. Eight publications included patients with functional class NYHA I, 10 publications included NYHA II, 11 included NYHA III and five included NYHA IV patients. Most patients belonged to NYHA III group, while in four studies the functional class of HF patients was not mentioned.

In most of the studies included in this review, biomarker levels between HF patients and controls were compared, and differences were found to be statistically significant, with one exception. No statistically significant difference was found in the sAA levels between controls and HF patients as reported by Suska et al. [36]. The levels of all biomarkers studied were found to be higher in HF patients compared to controls, except for salivary amylase that was found to be decreased in HF patients in one study [26], and sodium and chloride that had lower saliva concentrations in HF patients in another study [40]. In addition, one study included only HF patients (no controls) and studied the correlation between serum and salivary concentrations for specific biomarkers. Only a weak correlation was found for serum–salivary IL-6, while no correlation was found for serum–salivary BNP and IL-10 [29].

Interestingly, although natriuretic peptides have been officially incorporated in the diagnostic algorithm of HF [1], until the period that our research was conducted there were only three publications measuring salivary natriuretic peptides [29,30,35]. In all these studies, higher natriuretic peptide levels were found in the saliva in HF patients compared to controls, while no correlation was found between saliva and plasma concentrations. However, these studies were of small size and thus safe conclusions cannot be drawn. Larger studies are needed to validate the clinical importance of the saliva values of natriuretic peptides as well as their correlation to plasma levels.

Salivary cortisol has also been examined in three studies and although a non-specific index of HF, it should be considered, together with natriuretic peptides, the most extensively studied salivary biomarker in HF. Salivary cortisol was found to be increased in HF patients [38] and, more importantly, its levels were associated with patient outcomes. High salivary cortisol levels predicted decreased event-free survival [34], while high evening levels predicted increased mortality risk [32]. Another HF biomarker studied in saliva was Gal-3 which has been measured in two studies that showed higher levels in HF patients compared to controls, a moderate correlation between saliva and serum levels [33], and an association with patient outcomes. Salivary Gal-3 levels were associated with risk of cardiovascular death or hospitalization [28].

Data from two studies on salivary amylase and salivary alpha amylase have not been very promising [26,36]. A difference between HF patients and controls has only been shown for salivary amylase (lower levels for HF patients) [26], while both studies were limited by small size and large inter- and intra-subject variation. The results of these studies have put the potential value of salivary amylase or sAA as a HF biomarker into question. Lactate, a biomarker used frequently in acute HF [1], may be useful in HF monitoring as its salivary levels have been shown to correlate with NYHA class in one study [27]. Similarly, indices of oxidative stress such as salivary levels of 8-isoPGF2α and 8-epiPGF2α have been found to correlate with markers of HF severity such as serum NT-proBNP levels, NYHA class or LVEF [27,37] and may thus prove to be useful for monitoring of HF progression. UA has also shown some ability to assess functional status, in addition to discriminating between HF patients and controls [27].

CRP, a classical biomarker of inflammation, has been examined in saliva in a single study, but has not been associated with HF severity [29]. In the same study [29], other biomarkers such as IL-6, IL-10 and BNP were also studied but were not found to be useful for HF management. Interestingly, visible oral inflammation was the only predictor of HF severity in this study. A cytokine that was found to have a potential value in diagnosis and monitoring of HF was endothelin. However, this was only reported in a single study of limited size [39] and this finding needs to be confirmed in larger studies. Furthermore, S10A7, a protein of unknown pathophysiological significance for HF, was demonstrated to discriminate between HF and control subjects in an elegant study of saliva proteomic analysis [31]. Further studies are needed to investigate the pathogenetic role of S10A7 in HF and the extent of its clinical usefulness.

Finally, a study dated back in 1950 had assessed the salivary levels of the sodium, chloride and potassium [40]. The concept of measuring electrolytes in saliva is very interesting, especially since these are very frequently evaluated in HF patients in order to assess patients’ response to treatment. However, these biomarkers have been studied in one study only, and over 70 years ago, so more evidence is needed to recommend on their predictive value as HF biomarkers. Furthermore, salivary urea and creatinine concentrations, if they could be measured, would be proven to be very useful, especially for therapy monitoring in HF.

In summary, various salivary biomarkers have shown to be of value in the management of HF (Table 2). More specifically, cortisol and Gal-3 have shown to be useful for HF prognosis, natriuretic peptides, endothelin, Gal-3, amylase and S10A7 for HF diagnosis, while BNP, endothelin, cortisol, UA, 8-isoPGFα, 8-epiPGFα, lactate and electrolytes have been used for HF monitoring of severity progression. Regarding the correlation between saliva and serum concentrations of biomarkers, this has not been extensively studied (Table 2). A weak to moderate correlation was found only for endothelin [39], Gal-3 [33], CRP and IL-6 [29], while no correlation was found for NT-proBNP [35], BNP and IL-10 [29]. However, it should be emphasized that all these studies were small and larger studies should be done to clarify this.

Multiple biomarker assessment in HF patients for diagnostic and prognostic information is the pursuit of modern research in HF, and the saliva biomarkers studied so far aim towards that direction (Table 2). Also towards that direction, a research project is ongoing, aiming to create a novel point-of-care device that will be able to measure multiple saliva biomarkers (NT-proBNP, cortisol and possibly also TNF–α and IL-10) in HF patients and non-HF patients with risk factors such as obesity or hypertension. A software platform, based on machine learning techniques, might allow the extraction of patterns and rules towards efficient HF diagnosis and therapy monitoring in real time [64]. The use of saliva that is easy to acquire in any setting, in combination with a point-of-care device, are expected to render HF diagnosis and therapy monitoring feasible in primary health care or even at home, leading to a great cost reduction both for the health system and the patients.

## 5. Conclusions

Only few salivary biomarkers have been studied so far in HF patients. Natriuretic peptides are the most commonly used plasma biomarkers in the management of HF and their saliva levels have shown promising results, mainly for HF diagnosis. However, the role of salivary natriuretic peptides in the management of HF needs to be established in larger studies. Other biomarkers of interest in HF, such as Gal-3, endothelin and amylase, have shown a limited ability to discriminate between HF and non-HF patients, while cortisol, endothelin, UA, 8-isoPGFα, 8-epiPGFα, lactate and electrolytes have shown a limited ability to assess changes in clinical progression of HF patients. Of all those biomarkers, only salivary Gal-3 and cortisol have been associated with prognosis in HF patients. TNF-α has been identified in detectable concentrations in saliva but has been studied only in patients with oral cancers and inflammations. The correlation between saliva and serum concentrations has been little studied and only a weak to moderate correlation has been shown for endothelin, Gal-3, CRP and also IL-6. Whether the salivary levels of these biomarkers may play a significant role in the management of patients with HF needs to be investigated in future studies.

Assessment of a combination of several saliva biomarkers could potentially prove to be a more effective approach, especially if their measurement can be done accurately, easily, and fast. Despite the obvious practical benefits in using saliva samples, further research is required to overcome obstacles related to collection, storage, and analysis in order to facilitate the use of saliva biomarkers in everyday clinical practice. Especially in the era of the COVID-19 pandemic, saliva samples should be treated with great care and in accordance with existing international medical guidelines.

## Figures and Tables

**Table 1:** Studies included in the review, arranged by year of publication (descending).

Author	Year	Journal	*n* (HF)	Biomarker	Profile of HF Patients	Main Results
Klimiuk et al. [26].	2020	J Clin Med	50	Amylase, UA	LVEF ≤35%, NYHA II (*n* = 33)—III (*n* = 17), variable etiology	Salivary Amylase secretion, concentration and activity were decreased in the HF compared to the matched control group, indicating secretory dysfunction of salivary glands in HF. Salivary UA concentration was significantly higher in non-stimulated saliva of NYHA III compared to NYHA II patients. In simulated saliva, the activity of UA was significantly higher in HF patients compared to controls.
Ghimenti et al. [27].	2020	Sci Rep	44	8-isoPGF_2α_, Lactate, UA	Acute hospitalized HF, variable etiology and LVEF, NYHA I-IV	Salivary lactate and 8-isoPGF2α were strongly correlated with NT-proBNP. There was a significant decrease at discharge (*p* < 0.01 and *p* = 0.02 respectively), suggesting a relationship between salivary levels and clinical conditions during hospitalization. Only lactate was correlated (positively) to NYHA class.
Zhang et al. [28].	2019	Clin Res Cardiol	105	Gal-3	HFrEF, NYHA I-III, variable etiology	HF patients with Gal-3 concentrations of >172.58 ng/mL demonstrated a higher cumulative risk of either cardiovascular death or hospitalization compared to those with lower levels (*p* < 0.05). In HF patients, salivary Gal-3 was a predictor of the primary endpoint even after adjustment for covariates (co- morbidities, chronic obstructive pulmonary disease, past history of HF, medication, presence of implantable cardioverter-defibrillator).
Dekker et al. [29].	2017	Biol Res Nurs	75	BNP, IL-6, IL-10, CRP	HFpEF or HFrEF, NYHA I-III, variable etiology	Moderate correlation was found for serum–salivary CRP, weak correlation for serum–salivary IL-6, and no correlations for serum–salivary BNP and IL-10. The Bland–Altman test showed good agreement between saliva and serum for all biomarkers. As serum concentrations increased, salivary measures underestimated serum levels. No biomarkers were associated with NYHA class.
Joharimoghadam et al. [30].	2017	Kardiol Pol	70	BNP	HFrEF, LVEF 25 ± 3%	Salivary BNP levels were higher in admitted HF (*p* < 0.001) and outpatient HF patients (*p* = 0.02) compared to the control group. Salivary BNP may be useful in the diagnosis and follow-up of patients with HF, especially in emergencies.
Zhang et al. [31].	2017	Theranostics	36	S10A7	NYHA I-IV	Statistically significant differences (*p* < 0.05) were found between NYHA III/IV HF patients and controls.
Hammer et al. [32].	2016	Int J Cardiol	229	Cortisol	LVEF < 40%, NYHA III/IV, variable etiology	In univariate and multivariable models, the mortality risk of patients with the highest evening salivary cortisol was significantly increased, suggesting that associations of high evening salivary cortisol and increased mortality were independent of disease severity: crude HR 3.33 (*p* = 0.003), adjusted (for age, sex, NYHA class, and NT-proBNP) HR 2.49 (*p* = 0.047). Evening salivary cortisol was found to be the best predictor of mortality.
Zhang et al. [33].	2016	J Clin Pathol	63	Gal-3	HFrEF, hospitalized or outpatients	Gal-3 concentrations were significantly elevated in saliva and serum of HF patients compared with controls (*p* < 0.001 and *p* < 0.0001, respectively). A moderate correlation (r = 0.4, *p* < 0.01) was found between serum and salivary levels. No differences among NYHA classes. Larger multi-centre clinical trials are needed before salivary Gal-3 can be implemented in a clinical setting.
Alhurani et al. [34].	2014	SAGE Open Medicine	81	Cortisol	HFpEF or HFrEF, NYHA I-IV, variable etiology	Salivary cortisol was a significant predictor of 6-month cardiac event-free survival in HF patients (unadjusted for covariates, *p* = 0.05). Stress and NYHA class were not significant predictors of salivary cortisol level *p* = 0.32 and *p* = 0.5 respectively).
Foo et al. [35].	2012	PLoS ONE	45	NT-proBNP	HFrEF, NYHA III	Saliva NT-proBNP was higher in HF patients compared to controls. Saliva concentrations were more than 200-fold lower than plasma. The salivary NT-proBNP had a sensitivity of 82.2% and specificity of 100%, positive predictive value of 100% and negative predictive value of 83.3%, with an overall diagnostic accuracy of 90.6%.
Suska et al. [36].	2012	J Clin Exp Cardiol	24	sAA	NYHA I-III	No statistically significant difference in sAA levels was found between HF patients and controls. A strong tendency of higher morning values in patients was found, especially if measurements were done within 30 min after awakening. There was a strong inter- and intra-subject variation and a small number of participants. All HF patients were on b-blockers that are known to reduce the sAA levels.
Wolfram et al. [37].	2005	Eur J Heart Fail	40	8-epiPGF2α	Dilated (*n* = 20) and Ischemic cardiomyopathy (*n* = 20), HFpEF and HFrEF	8-epiPGF2α levels were correlated positively with NYHA class and negatively with LVEF, while they were significantly higher in patients with ischemic and dilated cardiomyopathy compared to controls and patients with coronary heart disease (*p* = 0.001).
Jekell et al. [38].	2004	Eur J Heart Fail	27	Cortisol	HFpEF or HFrEF, NYHA II-III, variable etiology	HF patients had higher morning levels of free cortisol than controls (*p* = 0.0002).
Denver et al. [39].	2000	Lancet	44	Endothelin	Chronic HFrEF, NYHA I-IV, variable etiology	Salivary endothelin concentrations were raised 2–to-6-fold in HF vs. controls (*p* = 0.005) and could detect HF with a sensitivity of 63% and specificity of 92%. A positive correlation between salivary and plasma endothelin concentrations was found in HF patients (*p* = 0.032). Endothelin levels were not significantly correlated to NYHA class when controls were excluded from analysis.
White et al. [40].	1950	J Clin Invest	27	Sodium, Chloride, Potassium	Congestive HF	Congestive HF was associated with lower sodium, lower chloride, and higher potassium concentrations in saliva compared to controls. Saliva of subjects on salt-poor diets did not show significant differences in electrolyte concentrations between HF patients and controls. No relationship in electrolyte concentrations between serum and saliva was found.

**Table 2:** Saliva biomarkers that have shown a potential role in HF management.

Author [Reference]	Year	Saliva Biomarker	Range (In Total Study Population)	Correlation between Saliva and Serum Levels	Clinical Usefulness
Foo et al. [35].	2012	NT-proBNP	18.3–748.7 pg/mL	No correlation found	Diagnosis
Joharimoghadam et al. [30].	2017	BNP	4.96–6.85 ng/L	Not assessed	Diagnosis Monitoring
Denver et al. [39].	2000	Endothelin	0.50–23.56 fmol/mL	r = 0.536, *p* = 0.032	Diagnosis Monitoring
Zhang et al. [33].	2016	Gal-3	2.8–2510 ng/mL	r = 0.4, *p* < 0.01	Diagnosis
Zhang et al. [28].	2019	Gal-3	16.04–869.3 ng/mL	Not assessed	Prognosis (association with cardiovascular death or hospitalization)
Zhang et al. [33].	2017	S10A7	Not assessed	Not assessed	Diagnosis
Hammer et al. [32].	2016	Cortisol	0.08–1.28 ng/mL	Not assessed	Prognosis (association with mortality)
Alhurani et al. [34].	2014	Cortisol	0.09–0.55 μg/dL	Not assessed	Prognosis (association with cardiac event free-survival)
Jekell et al. [38].	2004	Cortisol	15–23 nmol/L	Not assessed	Monitoring
Klimiuk et al. [26].	2020	Uric Acid	0.5818–0.862 ng/mL	Not assessed	Diagnosis, Monitoring
Ghimenti et al. [27].	2020	8-isoPGF_2α_	25–60 pg/mL	Not assessed	Monitoring
Wolfram et al. [37].	2005	8-epiPGF2α	43–111 pg/mL	Not assessed	Diagnosis, Monitoring
Ghimenti et al. [27].	2020	Lactate	190–3790 mmol/L	Not assessed	Monitoring
Klimiuk et al. [26].	2020	Amylase	0.12–0.21 μmol/mg	Not assessed	Diagnosis (lower levels in HF)
White et al. [40]	1950	Sodium Chloride Potassium	7.4–27.9 12.8–30.8 16.2–29.7 mEq/L	No correlation found	Monitoring

## Data Availability

All data supporting reported results can be found on PubMed. The details are provided in References.

## Abbreviations

8-isoPGF2α	8-isoprostaglandin F2α
8-epiPGF2α	8-epiprostaglandin F2α
BNP	brain natriuretic peptide
CRP	C-reactive protein
Gal-3	galectin-3
GDF-15	growth differentiation factor 15
HF	heart failure
HFmrEF	heart failure with mid-range ejection fraction
HFpEF	heart failure with preserved ejection fraction
HFrEF	heart failure with reduced ejection fraction
hsTn	high sensitivity troponin
IL-6	interleukin 6
IL-10	interleukin 10
LOD	limit of detection
LVEF	left ventricular ejection fraction
NT-proBNP	N-terminal pro-brain natriuretic peptide
NYHA	New York Heart Association
sAA	salivary alpha amylase
S100A7	protein S100-A7
ST2	soluble interleukin 1 receptor-like 1
TNF-α	tumor necrosis factor-α
UA	uric acid
